# Developing Effective Frameworks for Large Language Model–Based Medical Chatbots: Insights From Radiotherapy Education With ChatGPT

**DOI:** 10.2196/66633

**Published:** 2025-02-18

**Authors:** James C L Chow, Kay Li

**Affiliations:** 1 Department of Medical Physics Princess Margaret Cancer Centre University Health Network Toronto, ON Canada; 2 Department of Radiation Oncology Temerty Faculty of Medicine University of Toronto Toronto, ON Canada; 3 Department of English Faculty of Arts and Science University of Toronto Toronto, ON Canada

**Keywords:** artificial intelligence, AI, AI in medical education, radiotherapy chatbot, large language models, LLMs, medical chatbots, health care AI, ethical AI in health care, personalized learning, natural language processing, NLP, radiotherapy education, AI-driven learning tools

## Abstract

This Viewpoint proposes a robust framework for developing a medical chatbot dedicated to radiotherapy education, emphasizing accuracy, reliability, privacy, ethics, and future innovations. By analyzing existing research, the framework evaluates chatbot performance and identifies challenges such as content accuracy, bias, and system integration. The findings highlight opportunities for advancements in natural language processing, personalized learning, and immersive technologies. When designed with a focus on ethical standards and reliability, large language model–based chatbots could significantly impact radiotherapy education and health care delivery, positioning them as valuable tools for future developments in medical education globally.

## Introduction

**Figure 1:**
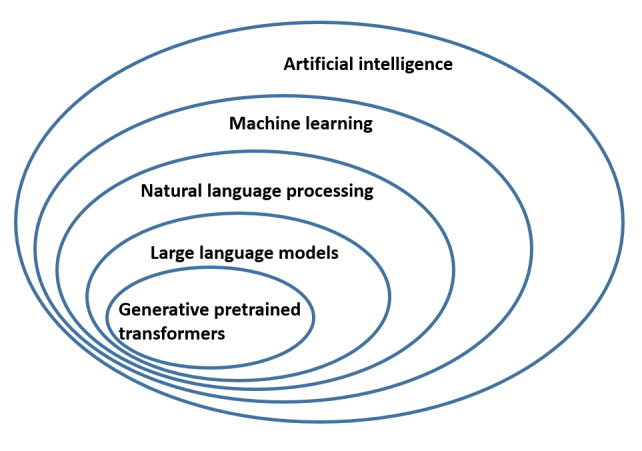
Relationships between key concepts in artificial intelligence, including machine learning, natural language processing, large language models, and generative pretrained transformers.

The integration of artificial intelligence (AI) in health care has rapidly evolved, with large language models (LLMs) such as ChatGPT at the forefront of this change [1-3]. These models, trained on vast datasets, have shown remarkable potential in various domains, including health care, by understanding and generating human-like text [4]. Unlike traditional rule-based chatbots, LLM-based systems can engage in complex conversations, answer medical queries, assist in symptom checking, and provide personalized health advice [5]. This capability is largely due to their training on extensive medical literature and patient data [6], which allow them to access and synthesize information in ways that were previously unimaginable [7]. The relationship between AI, machine learning, natural language processing (NLP), LLMs, and generative pretrained transformers is shown in Figure 1.

The deployment of LLM-based chatbots in health care is driven by the growing demand for accessible information and the need to alleviate the burden on health care systems [8]. These chatbots operate 24-7, providing immediate responses to patient inquiries; enhancing patient education; and supporting telemedicine, mental health, and chronic disease management [9-13]. However, their integration also presents challenges, such as ensuring the accuracy of information, avoiding biased responses, and safeguarding patient privacy [14].

Radiotherapy is a specialized field within oncology that demands precise and accurate information for effective treatment planning and education [15]. Unlike general medical topics, it involves complex concepts such as dose distribution, radiation physics, and the biological effects of ionizing radiation. Inaccurate information can lead to severe consequences, including inadequate treatment or harm to patients, making reliable dissemination critical [16].

Radiotherapy education serves 3 primary groups: patients, radiation staff, and the public. Patients require clear and empathetic guidance to understand their treatment, manage expectations, and make informed decisions, improving adherence and outcomes [17]. For health care professionals, education focuses on advanced training in treatment planning, safety protocols, and technological advancements to ensure high standards of care [18]. Public education, meanwhile, aims to demystify radiotherapy, reduce stigma, and promote awareness of its benefits, fostering trust in cancer treatment systems [19]. Providing accurate and ethically shared information across these groups is vital to ensure safety, informed decision-making, and the effective application of radiotherapy.

This Viewpoint explores a framework for integrating LLM-based chatbots in radiotherapy education, designed to ensure accuracy, reliability, and ethical integrity. The framework incorporates 3 key components: a controlled and curated database, a robust quality control system, and continuous monitoring mechanisms [20]. The curated database includes only verified and relevant medical literature and patient data, ensuring that the chatbot’s responses are accurate and up to date. The quality control system involves regular audits, database updates, and algorithms to identify and correct inaccuracies or biases. Continuous monitoring tracks the chatbot’s performance, enabling real-time adjustments and improvements [20]. By addressing challenges such as misinformation and patient privacy, this framework establishes a trustworthy and effective tool in radiotherapy education. Furthermore, it sets a new standard for AI-driven tools in specialized health care education by focusing on accuracy, reliability, and ethical integrity [20,21].

Ultimately, the ethical design and use of LLM-based medical chatbots involve collective efforts from policy makers and governments to maximize benefits while mitigating risks such as data privacy breaches and algorithmic bias [22].

## Current State of Medical Chatbots

### Development and Implementation of LLM-Based Chatbots

The development and implementation of LLM-based chatbots in health care have marked a significant evolution from earlier chatbot technologies. Unlike traditional rule-based systems that operated within a limited scope, LLM-based chatbots such as those powered by OpenAI’s ChatGPT have demonstrated the ability to understand and generate natural language with remarkable fluency and contextual awareness [23]. This advancement has led to a broad range of applications in health care, where these chatbots are being used to support patient interaction, provide medical information, assist with diagnosis, and streamline administrative tasks. A review of current LLM-based medical chatbots reveals a diversity of functionalities tailored to various health care needs [24]. One prominent example is ChatGPT itself, which has been adapted for use in several health care settings. ChatGPT can engage in detailed conversations with patients, answering questions about symptoms, explaining medical conditions, and providing guidance on the next steps in care. Its ability to understand nuanced queries and generate contextually appropriate responses has made it a valuable tool for preliminary consultations, particularly in telemedicine where immediate access to health care professionals may be limited [25]. Another notable use case is the deployment of LLM-based chatbots for chronic disease management [13]. These chatbots are designed to assist patients in managing conditions such as diabetes, hypertension, and asthma by providing personalized advice, reminding them to take medication, and offering lifestyle recommendations based on their medical history and real-time data inputs; for example, some LLM-based chatbots integrate with wearable devices to monitor patient vitals and deliver timely interventions, improving adherence to treatment plans and potentially reducing the need for emergency care [26]. In addition to patient-facing applications, LLM-based chatbots have been implemented to support health care professionals. These chatbots can function as web-based assistants, helping clinicians with tasks such as documentation, coding, and accessing up-to-date medical literature; for instance, some systems are designed to summarize patient records, extract relevant clinical information, and even suggest differential diagnoses, thereby enhancing the efficiency of clinical workflows [27]. In research settings, LLM-based chatbots are used to assist in data analysis, generate research hypotheses, and streamline the process of literature review by quickly summarizing large volumes of academic papers.

The flexibility and scalability of LLM-based chatbots have also enabled their adoption in mental health support. Chatbots such as Woebot [28] and Wysa [29] use LLMs to engage in therapeutic conversations, offering cognitive behavioral therapy techniques, mood tracking, and crisis intervention. These tools provide users with immediate access to mental health support, which is particularly valuable in regions with limited access to mental health professionals or during times when in-person therapy is not feasible. While the functionalities and use cases of LLM-based chatbots in health care are vast, the implementation of these tools is not without challenges. Ensuring the accuracy and reliability of the information provided by these chatbots is crucial, especially in scenarios where they are used for diagnosis or treatment recommendations. Moreover, the ethical implications of using AI in patient care, particularly in terms of data privacy and informed consent, require careful consideration [30]. Despite these challenges, the ongoing development and refinement of LLM-based chatbots continue to push the boundaries of what is possible in health care, offering promising avenues for improving patient care, enhancing health care delivery, and supporting the work of health care professionals.

### Strengths and Limitations

The implementation of LLM-based chatbots in health care, particularly in specialized fields such as radiotherapy, offers both significant strengths and notable limitations. As these advanced AI-driven tools become more integrated into medical practice, it is crucial to understand their advantages and challenges to optimize their use while mitigating potential risks.

### Strengths

One of the primary strengths of LLM-based chatbots is their ability to process and generate natural language with a high degree of fluency and context sensitivity. This capability allows them to engage in detailed and nuanced conversations with both patients and health care professionals, making complex medical information more accessible and understandable. In radiotherapy, where patients often face intricate treatment regimens and technical jargon, an LLM-based chatbot can break down complex concepts into simpler terms, enhancing patient comprehension and engagement. This improved communication can lead to better patient adherence to treatment plans and a more informed patient population, which is essential for the success of radiotherapy [17]. Another significant advantage of LLM-based chatbots is their scalability and availability. These chatbots can provide consistent, round-the-clock support, making them particularly valuable in settings where access to health care professionals may be limited. In radiotherapy, where timely information is critical, a chatbot can offer immediate answers to patient queries, provide pretreatment education, and even guide patients through posttreatment care [31]. This constant availability helps reduce the burden on health care providers, allowing them to focus on more complex cases and personalized care. Furthermore, LLM-based chatbots can be regularly updated with the latest medical research and guidelines, ensuring that the information they provide remains current. In a field such as radiotherapy, where treatment protocols and technologies are continuously evolving, this ability to quickly incorporate new knowledge is a significant strength. It enables the chatbot to serve as a reliable resource for both patients and health care professionals, supporting evidence-based practice and reducing the likelihood of outdated or incorrect information being disseminated [32].

### Limitations

Despite their strengths, LLM-based chatbots also present several limitations, particularly in specialized medical fields such as radiotherapy. One of the most pressing challenges is ensuring the accuracy and reliability of the information provided by these chatbots [20]. While LLMs can generate text that seems plausible and coherent, they may occasionally produce incorrect or misleading information, especially when faced with highly specialized or uncommon queries. In radiotherapy, where precise details about treatment options, dosimetry, and potential side effects are crucial, even small inaccuracies can have serious consequences for patient care. Another limitation is the potential for LLM-based chatbots to oversimplify complex medical information. While simplifying language is essential for patient comprehension, there is a risk that crucial nuances may be lost, leading to misunderstandings or incomplete knowledge. In radiotherapy, where patients need to fully understand their treatment options, the risks, and the benefits, this oversimplification could impact their ability to make informed decisions [3]. Ethical considerations also pose significant challenges for the implementation of LLM-based chatbots in health care. Issues related to patient privacy, data security, and the potential for bias in the AI’s responses are critical concerns. In radiotherapy, where patients are dealing with life-altering decisions and sensitive health information, ensuring that the chatbot operates within strict ethical guidelines is essential. There is also the challenge of maintaining transparency in how these chatbots operate and ensuring that patients are fully aware that they are interacting with an AI, not a human health care provider. Moreover, the reliance on LLM-based chatbots could potentially lead to an overdependence on AI at the expense of human interaction. In specialized fields such as radiotherapy, the human touch is often crucial for providing emotional support and addressing the psychosocial aspects of care. While chatbots can provide information and support, they cannot replace the empathy and personalized care that human health care providers offer [33].

### Summary

Table 1 summarizes the key advantages and challenges associated with the deployment of LLM-based chatbots in health care, particularly in the context of radiotherapy, where precision, ethical considerations, and human interaction are critical. It is seen that while LLM-based chatbots offer significant strengths, including enhanced communication, scalability, and up-to-date information, they also come with limitations that must be carefully managed. In specialized fields such as radiotherapy, where there is a need for compassion in addition to accurate information as the users may be in stressful situations, the deployment of these chatbots requires a well–thought-out framework that maximizes their benefits while addressing their inherent challenges.

**Table 1:** Strengths and limitations of large language model–based chatbots in specialized medical fields such as radiotherapy.

Aspect	Strengths	Limitations
Natural language processing	Fluent, context-aware communication Simplifies complex medical information for better patient understanding	Risk of generating incorrect or misleading information Potential oversimplification of complex concepts, leading to misunderstandings
Availability and scalability	24-7 availability for patient and health care provider support Reduces the burden on health care professionals by handling routine queries	Overdependence on AI^a^ could reduce human interaction, which is vital in emotionally supportive care, particularly in radiotherapy
Up-to-date information	Can be regularly updated with the latest research and guidelines, ensuring that the information provided is current	Challenges in maintaining the accuracy of specialized information, especially as new research emerges and medical knowledge evolves
Patient engagement	Enhances patient education and engagement through personalized, accessible information Supports informed decision-making in complex treatments	Risk of inadequate personalization, potentially leading to generic advice that does not fully meet individual patient needs
Ethical considerations	Can be programmed to follow ethical guidelines, ensuring responsible dissemination of medical information	Issues related to patient privacy, data security, and AI bias Potential lack of transparency in AI operations, affecting trust
Efficiency in health care	Improves efficiency by assisting in documentation, coding, and access to medical literature for health care providers	Dependency on chatbot accuracy for clinical tasks might introduce errors if the chatbot provides incorrect or incomplete information

^a^AI: artificial intelligence.

### Impact on Patient Education and Health Care Delivery

#### Influence on Patient Education

**Figure 2:**
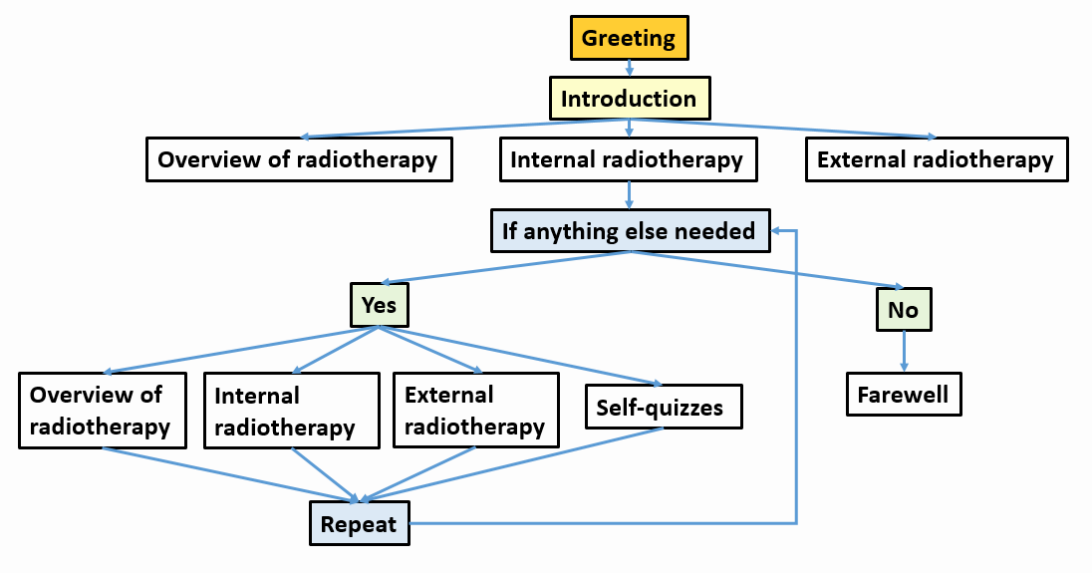
Typical dialogue flowchart for a chatbot used in radiotherapy education.

LLM-based chatbots play a significant role in enhancing patient education by making complex medical information more accessible. In specialized fields such as radiotherapy, where patients must understand intricate treatment protocols and potential side effects, chatbots provide a valuable resource for simplifying and clarifying these concepts. By translating technical jargon into clear language, chatbots help patients grasp the fundamentals of their treatment, which can be crucial for their engagement and adherence. Moreover, chatbots can offer tailored educational content based on individual patient profiles; for example, a chatbot might provide specific information about the type of radiotherapy a patient is receiving, potential side effects relevant to their case, and strategies for managing these side effects [31,34]. This personalized approach ensures that patients receive relevant information that directly pertains to their situation, enhancing their understanding and preparedness for their treatment journey. Figure 2 shows a typical dialogue flowchart for a chatbot in radiotherapy education.

#### Impact on Decision-Making

In addition to enhancing patient education, LLM-based chatbots significantly influence patient decision-making. By providing timely and accurate information, these tools empower patients to make informed choices about their treatment options; for instance, a chatbot can help patients compare different radiotherapy techniques, discuss the potential benefits and risks of each option, and address any concerns they may have. This support is especially valuable in radiotherapy, where patients often face complex decisions about their care [32]. The chatbot’s ability to offer evidence-based information and answer questions in real time helps reduce uncertainty and anxiety, allowing patients to engage more effectively in their treatment planning. Furthermore, by facilitating a deeper understanding of their condition and treatment options, chatbots contribute to a more collaborative decision-making process between patients and their health care providers.

#### Enhancement of Health Care Delivery

The implementation of LLM-based chatbots also has a notable impact on the overall delivery of health care. These tools streamline administrative tasks, such as appointment scheduling, medication reminders, and follow-up care, thus freeing up health care professionals to focus on more complex patient interactions [35]. In radiotherapy, where treatment regimens are often intensive and require careful management, chatbots can help monitor patient progress, manage treatment schedules, and provide reminders for follow-up appointments or adherence to prescribed protocols. In addition, chatbots can assist in triaging patient queries, directing them to appropriate resources or health care professionals based on the nature of their concerns [36]. This efficient handling of inquiries helps ensure that patients receive timely and relevant support, which can improve their overall experience and satisfaction with their care.

### Challenges in Deploying LLM-Based Medical Chatbots

#### Overview

Despite the promising advancements and benefits of LLM-based medical chatbots, their deployment in health care settings is accompanied by several significant challenges. These challenges span various critical areas, including the accuracy and reliability of the information provided, privacy and data security concerns, ethical considerations, and the potential for misinformation. Addressing these issues is essential to ensure that chatbots contribute positively to health care delivery while mitigating risks.

#### Accuracy and Reliability

Ensuring the accuracy and reliability of medical information provided by LLM-based chatbots is a critical challenge that affects their effectiveness and safety in health care settings. The complexity of medical knowledge and the rapid evolution of treatment protocols present significant hurdles in maintaining the precision of information delivered by these AI tools. One major challenge is the inherent nature of LLMs, which are trained on vast datasets that include a wide range of information sources. While this breadth of data allows chatbots to generate responses to a variety of queries, it also means that the information they provide can be inconsistent or outdated [37]. In the context of radiotherapy, where treatment guidelines and protocols are continually updated based on the latest research, ensuring that a chatbot reflects the most current and accurate information is crucial. Failure to do so can result in the dissemination of outdated or incorrect treatment recommendations, potentially compromising patient safety [20].

In addition, LLM-based chatbots rely on pattern recognition and probabilistic inference rather than deep, context-specific understanding [38]. This approach can sometimes lead to the generation of plausible but inaccurate answers, especially when dealing with rare or complex medical scenarios; for example, a chatbot might provide general information about radiotherapy but struggle with specifics related to individual patient conditions or less common treatment modalities [4]. This limitation highlights the need for robust validation processes to ensure that the chatbot’s responses are not only accurate but also relevant to the user’s specific context. Another aspect of accuracy and reliability involves the management of the chatbot’s knowledge base [39]. Regular updates and reviews are necessary to keep the information current and accurate. This process requires a continuous feedback loop from health care professionals who can assess and correct any inaccuracies or gaps in the information provided by the chatbot. Implementing such a system involves collaboration between AI developers and medical experts to ensure that the chatbot’s responses align with the latest evidence-based practices. Moreover, there is a challenge in balancing the chatbot’s ability to generate detailed responses with the need to provide clear and concise information [40]. Overly complex or technical explanations can be difficult for patients to understand, while overly simplified answers may omit critical details. Striking the right balance is essential to provide information that is both accurate and accessible to users with varying levels of medical knowledge.

#### Privacy and Data Security

Privacy and data security are paramount concerns when deploying LLM-based chatbots in health care, given the sensitive nature of patient information handled by these tools. Ensuring the protection of these data against unauthorized access and breaches is essential to maintaining patient trust and complying with regulatory requirements [19]. One of the primary challenges in safeguarding patient data involves ensuring compliance with privacy regulations such as the Health Insurance Portability and Accountability Act in the United States [41] or the General Data Protection Regulation in Europe [42]. These regulations mandate stringent measures for the collection, storage, and handling of personal health information. LLM-based chatbots must be designed to adhere to these regulations, incorporating robust data encryption methods, secure storage solutions, and strict access controls to protect sensitive patient information from breaches and unauthorized access. Data transmission security is another critical aspect. When chatbots interact with patients, the data exchanged—such as medical history, treatment details, and personal identifiers—must be encrypted to prevent interception by malicious actors. Implementing secure communication protocols, such as transport layer security, helps ensure that data transmitted between the chatbot and the user are protected from eavesdropping and tampering [43]. In addition, managing user consent and data use transparency is crucial for maintaining privacy. Patients must be informed about what data are being collected, how the data will be used, and their rights regarding their information. Clear consent mechanisms should be integrated into the chatbot’s design, allowing users to provide informed consent before any data collection occurs. This transparency helps build trust and ensures that patients are aware of how their data are being handled.

Another challenge is ensuring that data anonymization and deidentification practices are effectively applied. Even when data are stored or processed for purposes such as improving chatbot performance or training new models, it is essential to anonymize the data to prevent the identification of individual patients. Proper anonymization techniques reduce the risk of sensitive information being exposed in case of a data breach. Moreover, continuous monitoring and auditing of the chatbot’s security systems are necessary to identify and address potential vulnerabilities. Regular security assessments, including penetration testing and vulnerability scanning, help ensure that the chatbot’s infrastructure remains resilient against emerging threats. Promptly addressing any identified weaknesses is crucial for maintaining the overall security of patient data.

#### Ethical Considerations

##### Overview

The deployment of LLM-based chatbots in health care raises several ethical dilemmas that must be carefully addressed to ensure the responsible and fair use of AI. Key ethical concerns include AI decision-making processes, transparency, and accountability, each of which plays a critical role in maintaining trust and integrity in health care interactions [44]. To address these concerns, transparency can be enhanced by developing clear documentation that explains the chatbot’s decision-making processes, including data sources and logic. Bias detection and mitigation tools, such as fairness-aware algorithms, should be used to identify and correct biases in real time. Regular audits by independent third parties can ensure compliance with ethical standards.

User feedback mechanisms are essential for identifying and addressing ethical issues. Users should be able to report concerns or errors, which should be promptly reviewed and acted upon. In addition, clear guidelines for human oversight must be established, defining the roles of health care professionals in monitoring chatbot interactions. By incorporating these detailed suggestions, processes, and tools, we can better address ethical issues and promote the responsible use of AI in health care [18].

##### Long-Term Societal Impacts

The widespread adoption of ChatGPT and similar technologies could significantly reshape the job market, potentially leading to both job displacement and the creation of new opportunities, necessitating reskilling and upskilling programs to mitigate negative impacts. Moreover, AI technologies might exacerbate economic inequalities if their benefits are not equitably distributed, highlighting the need for policies that promote inclusive growth. The integration of AI into daily life could alter social interactions, reducing human-to-human contact and impacting social skills and relationships, thus requiring a balance between AI use and genuine human connection. These changes underscore the importance of robust ethical governance frameworks involving diverse stakeholders to ensure that AI development aligns with societal values and ethical principles, addressing emerging challenges and opportunities through continuous dialogue and adaptive regulation.

##### AI Decision-Making

One of the central ethical issues is the decision-making capability of AI systems. Unlike human health care providers who can apply clinical judgment and empathy, LLM-based chatbots operate based on algorithms and data patterns [45]. This raises questions about the extent to which these systems can make ethical decisions, especially in complex or nuanced medical scenarios; for instance, while chatbots can provide general information and support, they may lack the ability to fully understand the context of a patient’s situation or the ethical implications of certain recommendations [46]. Ensuring that AI-driven tools align with ethical guidelines and medical standards is essential to avoid potentially harmful outcomes.

##### Transparency

Transparency in AI decision-making is another critical ethical consideration. Patients and health care professionals need to understand how chatbots generate their responses and make recommendations. This includes clarity about the data sources and algorithms that drive the chatbot’s behavior. Without transparency, there is a risk that users might overestimate the chatbot’s capabilities or misinterpret its advice. Providing clear information about the chatbot’s operational mechanisms helps build trust and allows users to make informed decisions about how to use the tool effectively [47]. Furthermore, transparency extends to the disclosure of limitations and potential biases inherent in the AI system. Chatbots are trained on datasets that may reflect existing biases or incomplete information, which can affect the fairness and objectivity of their responses [48]. Openly communicating these limitations and actively working to mitigate bias are crucial for maintaining ethical standards and ensuring that the chatbot serves all users equitably.

##### Accountability

Accountability is a significant ethical issue concerning the use of LLM-based chatbots. Determining who is responsible for the chatbot’s recommendations and decisions is essential, particularly in cases where incorrect or harmful information is provided. Clear lines of accountability must be established, involving both the developers who create and maintain the chatbot and the health care providers who deploy it. This includes ensuring that there are mechanisms for addressing errors or issues that arise from the chatbot’s interactions with users [49]. In addition, it is important to have protocols in place for managing situations where the chatbot’s advice might lead to adverse outcomes [50]. This includes providing a way for users to report issues and seek redress, as well as ensuring that there are processes for continuous improvement based on feedback and incident analysis. Effective oversight and governance structures help ensure that ethical standards are upheld and that the chatbot contributes positively to patient care.

#### Potential for Misinformation

##### Overview

The potential for misinformation is a critical concern in the deployment of LLM-based medical chatbots. Given their reliance on vast datasets and sophisticated algorithms, these chatbots can inadvertently spread incorrect or misleading information, which poses significant risks to patient health and safety. As deep learning pioneer Geoffrey Hinton has noted, neural networks can share what they learn instantly [22]; therefore, any erroneous messages can spread instantaneously, making unwanted wide impact.

##### Sources of Misinformation

One key source of misinformation is the quality and accuracy of the training data used to develop the chatbot. LLMs are trained on diverse datasets that include information from various sources, some of which may be outdated, biased, or inaccurate. If a chatbot’s training data contain erroneous or misleading content, there is a risk that the chatbot will replicate and disseminate these inaccuracies in its responses. In fields such as radiotherapy, where precise and current information is crucial, the presence of outdated or incorrect data can lead to harmful consequences for patients relying on the chatbot for guidance [51]. Another potential source of misinformation is the chatbot’s ability to generalize information [52]. While LLM-based chatbots can handle a wide range of queries, they may not always be adept at distinguishing between general knowledge and specific, context-sensitive details. This limitation can lead to the generation of responses that are technically correct but fail to address the nuances of individual patient situations [53]; for example, a chatbot might provide general advice on radiation safety but miss specific recommendations tailored to a patient’s unique treatment plan.

##### Risks and Consequences

The spread of incorrect or misleading information by medical chatbots can have serious repercussions for patient care. Patients may make health-related decisions based on inaccurate information, which can result in inappropriate or ineffective treatments. In radiotherapy, where treatment decisions are complex and require careful consideration of numerous factors, relying on incorrect information can lead to suboptimal care or adverse outcomes. Moreover, misinformation can erode trust in health care technology. If patients or health care providers encounter inaccuracies or inconsistencies in the information provided by a chatbot, their confidence in the tool’s reliability and usefulness may be undermined. This loss of trust can diminish the chatbot’s effectiveness and impact its acceptance and integration into health care practices.

##### Mitigation Strategies

To mitigate the risks associated with misinformation, several strategies can be used [54]. Regular updates and maintenance of the chatbot’s knowledge base are essential to ensure that it reflects the most current and accurate information. Collaboration with medical experts and continuous validation of the chatbot’s responses help identify and correct inaccuracies. Implementing mechanisms for user feedback and reporting can also provide valuable insights into potential issues and facilitate prompt resolution. Furthermore, incorporating a system of checks and balances, such as providing disclaimers that emphasize the chatbot’s limitations and the need for professional medical consultation, can help manage user expectations. Ensuring that the chatbot directs users to seek advice from qualified health care professionals when necessary can prevent reliance on potentially flawed or incomplete information [55].

## Proposed Framework for a Resilient Medical Chatbot in Radiotherapy Education

### Database Management and Control

Effective database management is crucial for developing a resilient medical chatbot in radiotherapy education. The integrity of the chatbot’s responses depends heavily on the accuracy and timeliness of the information it accesses. To achieve this, a robust strategy for building and maintaining a controlled database is essential [56]. The first step in this strategy involves curating a comprehensive and authoritative database from verified medical sources, such as peer-reviewed journals, clinical guidelines, and expert consensus statements. This curated database should be dynamic, with automated systems in place for continuous updates. The proposed framework incorporates an AI-driven mechanism for real-time monitoring of new publications and guidelines, ensuring that the database remains current and reducing the risk of disseminating outdated or inaccurate information [57]. To implement this, automated updates could leverage web scraping tools or application programming interface integrations to collect and validate data against trusted sources. The database should use meta-tagging and hierarchical organization for efficient data management and retrieval. Meta-tagging can be achieved using NLP algorithms to assign contextual keywords, improving the chatbot’s ability to interpret and respond to user queries accurately. A hierarchical data structure, such as a tree- or graph-based model, can prioritize data by relevance and reliability, ensuring that the chatbot provides the most appropriate responses [58,59]. In addition, periodic audits of the database should be conducted to verify its accuracy and adherence to updated guidelines.

Maintaining control over the database is equally important. This involves setting strict protocols for data entry and modification, with access restricted to authorized personnel who are well versed in radiotherapy and medical education. Regular audits of the database content should be conducted to identify and rectify any inconsistencies or errors [57]. In addition, using version control systems can help track changes, allowing for the restoration of previous database states if needed, which further enhances the reliability of the chatbot. To ensure that the database remains resilient against emerging challenges such as misinformation or biased data, a layered review process should be integrated. This involves cross-referencing new data entries with multiple sources and using machine learning algorithms to detect and flag anomalies or conflicting information [60]. By implementing these strategies, the database management and control framework will serve as the foundation for a resilient medical chatbot capable of providing accurate, reliable, and up-to-date information in radiotherapy education.

### Quality Control System

A robust quality control system is vital for ensuring the reliability and effectiveness of a medical chatbot in radiotherapy education. The system must be designed to maintain the highest standards of accuracy, relevance, and trustworthiness in the chatbot’s content, which requires regular reviews, timely updates, and stringent validation processes [61]. The cornerstone of this quality control system is the implementation of a multitiered review process. This begins with the periodic assessment of the chatbot’s content by a panel of experts in radiotherapy and medical education. These experts should evaluate the chatbot’s responses for accuracy, clarity, and consistency with current clinical practices. Regularly scheduled reviews ensure that the content remains aligned with the latest advancements in radiotherapy and adheres to evolving educational standards [4].

In addition to expert reviews, the quality control system should include automated checks and balances. Machine learning algorithms can be deployed to continuously monitor the chatbot’s interactions, identifying patterns of errors or discrepancies in the responses [62]. These algorithms can flag potential issues for further human review, ensuring that errors are caught and corrected promptly. Automated processes should also include regular updates to the chatbot’s knowledge base, triggered by new research findings, clinical guidelines, or changes in medical protocols. Another critical aspect of the quality control system is the validation of the chatbot’s content before it goes live. This can be achieved through rigorous testing with simulated user interactions, covering a wide range of scenarios that the chatbot is likely to encounter. Feedback loops from these tests should be analyzed to refine the chatbot’s algorithms and content delivery mechanisms, ensuring that it provides accurate and contextually appropriate responses [63]. Finally, the quality control system must be adaptable, with mechanisms in place for continuous improvement. This includes incorporating user feedback to identify areas where the chatbot’s performance may be lacking and making necessary adjustments. Moreover, the system should be capable of responding to unforeseen challenges, such as the propagation of misinformation, and adapting to emerging trends in radiotherapy by swiftly updating the chatbot’s content and protocols [17].

### Monitoring and Feedback Loop

Establishing an effective monitoring and feedback loop is essential for the continuous improvement and reliability of a medical chatbot in radiotherapy education. This system ensures that the chatbot consistently meets user needs, adapts to new information, and addresses any issues that arise during its interactions. The foundation of this system is a comprehensive monitoring mechanism designed to track all chatbot interactions in real time. By recording user queries, responses, and outcomes, this monitoring system can identify patterns in user behavior and detect potential issues, such as inaccurate answers, misinterpretations, or gaps in the chatbot’s knowledge base [64]. Advanced analytics tools can be integrated to automatically flag problematic interactions for further review by the development team, enabling swift corrective actions. In addition to tracking interactions, the monitoring system should incorporate a robust feedback loop that allows users to provide direct input on the chatbot’s performance [65]. This feedback can be collected through postinteraction surveys, ratings, or optional comment fields, giving users the opportunity to highlight areas where the chatbot excels or falls short. Aggregating and analyzing this feedback offers valuable insights into the chatbot’s strengths and weaknesses, guiding future updates and enhancements.

To maximize the effectiveness of the feedback loop, it is crucial to establish a process for prioritizing and addressing the issues identified. A triage system can be implemented to categorize user feedback based on severity and frequency, ensuring that the most critical issues are addressed promptly [66]; for example, if multiple users report the same error or misunderstanding, this issue would be flagged as a high priority for immediate resolution. Less critical feedback, such as suggestions for improved phrasing or additional features, can be scheduled for consideration in future updates. Moreover, the feedback loop should be designed to foster continuous learning and adaptation. Regularly scheduled reviews of the chatbot’s performance, informed by user feedback and monitoring data, should be conducted to identify trends and areas for improvement. This iterative process allows the chatbot to evolve over time, enhancing its ability to provide accurate, reliable, and contextually relevant information in radiotherapy education.

### Ethical and Legal Safeguards

Implementing robust ethical guidelines and legal safeguards is paramount in developing a medical chatbot for radiotherapy education. These measures are critical to addressing concerns related to privacy, transparency, and accountability, ensuring that the chatbot operates within the highest ethical and legal standards. To begin with, the chatbot must be designed with stringent privacy protections. Given the sensitive nature of medical information, the chatbot should comply with all relevant data protection laws, such as the General Data Protection Regulation in Europe or the Health Insurance Portability and Accountability Act in the United States [19]. This involves implementing secure data encryption methods, anonymizing user interactions, and ensuring that any personal or health-related data collected during interactions are stored securely and used only for their intended purpose [67]. Access to these data should be strictly controlled, with clear protocols for who can view, modify, or delete the data, ensuring that user privacy is respected at all times. Transparency is another critical aspect of ethical chatbot design. Users must be fully informed about how the chatbot functions, including the sources of its information, the limitations of its advice, and the nature of its data collection practices. This can be achieved by providing clear and accessible information within the chatbot interface, such as disclaimers before interactions, a detailed frequently asked questions section, or links to privacy policies. In addition, the chatbot should be designed to clearly distinguish between general information and personalized advice, ensuring that users understand the context and limitations of the information they receive [68].

Accountability mechanisms are also essential to uphold ethical standards. The development and deployment of the chatbot should include a clear governance structure, where responsibilities for content accuracy, data management, and user interactions are well defined. Regular audits of the chatbot’s performance and adherence to ethical guidelines should be conducted, with the results made available to relevant stakeholders [69]. Furthermore, there should be a clear process for users to report any ethical concerns or grievances, and these issues should be addressed promptly and transparently. To further enhance ethical and legal safeguards, it is important to establish an oversight committee consisting of experts in ethics, law, and medical education [70]. This committee would be responsible for reviewing the chatbot’s operations, ensuring that it remains aligned with ethical principles and legal requirements. The committee should also be tasked with evaluating the impact of the chatbot on users, particularly in terms of potential biases, misinformation, or unintended consequences, and recommending corrective actions as needed. The proposed framework is summarized in Table 2.

**Table 2:** Summary of the proposed framework for a resilient medical chatbot in radiotherapy education. The key components, their essential elements, and their respective purposes in ensuring the chatbot’s accuracy, reliability, and ethical operation are outlined.

Component	Key elements	Purpose
Database management and control	Curated and dynamic database Automated updates Hierarchical data structure	Ensures that the chatbot has access to accurate, up-to-date, and relevant information
Quality control system	Multitiered expert review Automated checks Content validation	Maintains high standards of accuracy, relevance, and reliability in chatbot responses
Monitoring and feedback loop	Real-time interaction tracking User feedback integration Continuous improvement	Tracks chatbot performance, identifies issues, and incorporates user feedback for ongoing enhancement
Ethical and legal safeguards	Privacy protections Transparency measures Accountability mechanisms	Addresses concerns related to user privacy, ensures transparency, and upholds accountability

## Case Study: Application of the Framework in Radiotherapy Education

### Outlining the Process of Developing and Testing the Chatbot Using the Proposed Framework

**Figure 3:**
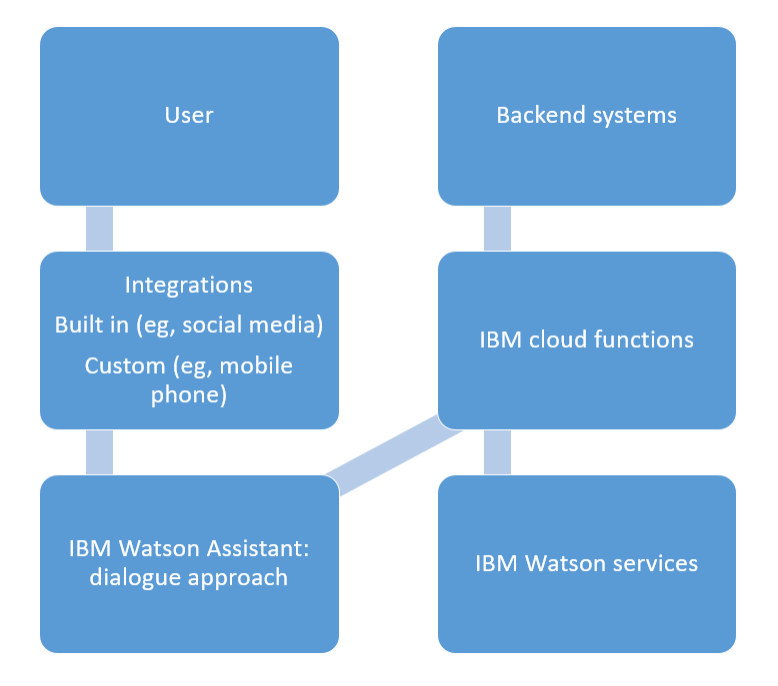
Architectural diagram showing how the chatbot is connected to the IBM Watson Assistant cloud platform.

The development of the radiotherapy education chatbot using the proposed framework commenced with a comprehensive needs assessment. This step involved engaging key stakeholders, including radiotherapy educators, clinical oncologists, medical physicists, and students, to identify the specific educational challenges and gaps that the chatbot aimed to address [32]. The focus was on creating a tool that could effectively supplement traditional education by providing accurate, accessible, and contextually relevant information on complex radiotherapy topics. Once the needs were clearly defined, the next phase involved designing the chatbot’s conversational architecture. The framework emphasized the integration of AI-driven NLP capabilities with a structured knowledge base specific to radiotherapy [71]. This phase required meticulous curation and validation of content, ensuring that the information was not only accurate but also aligned with the latest guidelines and research in the field. A collaborative approach was adopted, involving experts from various disciplines to ensure that the chatbot’s responses would be both scientifically robust and pedagogically sound [72]. The chatbot was then built using a hybrid approach that combined rule-based algorithms with machine learning models [73]. The rule-based components ensured that the chatbot could handle critical educational scenarios with precision, while the machine learning aspects allowed for more dynamic and flexible interactions. The integration with the IBM Watson Assistant cloud platform facilitated the deployment of advanced NLP algorithms, enabling the chatbot to understand and respond to user queries effectively [32]. Figure 3 shows the architectural diagram of a chatbot developed using the IBM Watson Assistant platform [74].

Testing the chatbot was an iterative process. Initial prototypes were subjected to rigorous testing in simulated environments, where various scenarios were presented to evaluate the chatbot’s performance in real time [75]. The testing focused on several key metrics: the accuracy of information, user engagement, response time, and the ability to handle unexpected or complex queries. Feedback from educators and students was crucial during this phase, providing insights into the chatbot’s usability and effectiveness in an educational setting. To ensure the reliability and safety of the chatbot, the testing also included a comprehensive evaluation of ethical considerations. The framework’s built-in mechanisms for privacy protection, data security, and bias mitigation were scrutinized to prevent potential harm. The chatbot was tested for its adherence to ethical standards through a Delphi study involving international experts, ensuring that it met global expectations for AI-driven educational tools [76].

The final phase involved pilot-testing the chatbot in real educational settings, specifically within radiotherapy courses. These studies aimed to evaluate both the chatbot’s technical performance and its educational impact. Observations from the pilot phase revealed notable improvements in student comprehension and engagement; for example, the chatbot, called RT Bot, was deployed on a website accessible by invitation and introduced during workshops and conferences to test its functionality. Participants were invited to complete a survey evaluating the chatbot’s functionality and content. This survey, conducted with 60 participants, aimed to gather statistical insights and feedback for improving the chatbot in the future. The results, summarized in Table 3 [32], highlight varying levels of user satisfaction with RT Bot during its testing phase. The assessment used a scale ranging from 1 (lowest satisfaction) to 5 (highest satisfaction). The data indicate that a substantial 70% (42/60) of the respondents rated the content’s helpfulness, understandability, and reading duration as above average.

**Table 3:** Users’ satisfaction ratings regarding the contents of RT Bot (N=60) [32].

Degree of satisfaction (ranging from 1=lowest to 5=highest)	Helpfulness of content, n (%)	Understandability of content, n (%)	Reading duration, n (%)
1	3 (5)	0 (0)	0 (0)
2	0 (0)	9 (15)	3 (5)
3	15 (25)	9 (15)	15 (25)
4	24 (40)	24 (40)	18 (30)
5	18 (30)	18 (30)	24 (40)

Specific feedback highlighted areas for improvement, such as the need for more intuitive methods to restart conversations and tailored hints for users with diverse backgrounds when answering radiation safety questions. These insights enabled developers to refine the chatbot’s adaptability and responsiveness. Data collected from the pilot tests demonstrated a measurable positive effect on learning outcomes, as students achieved higher quiz scores and expressed increased confidence in applying radiation safety principles. The feedback loop established during this phase ensured that the chatbot evolved into a robust, user-centered educational tool, ready to support radiotherapy training effectively [34,77,78].

**Figure 4:**
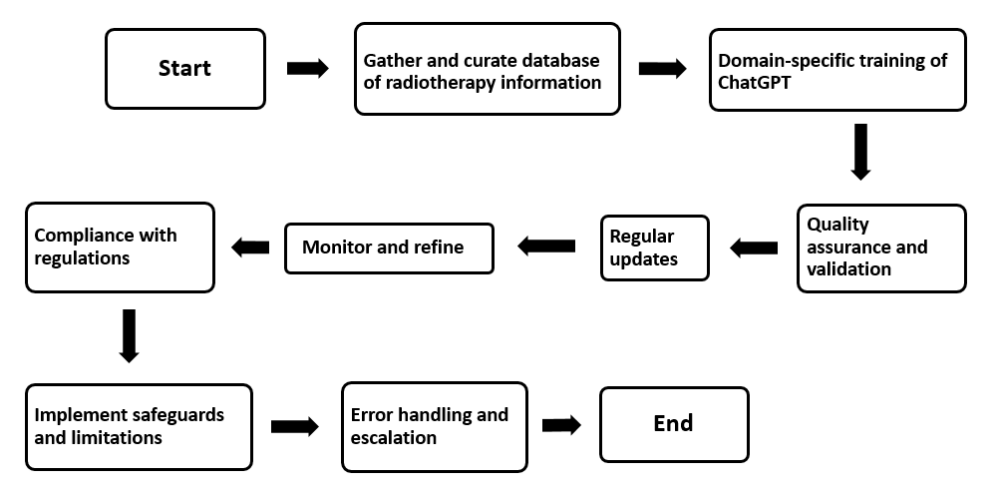
Flowchart providing a sequential representation of the steps involved in creating, maintaining, and improving a medical chatbot based on the large language model ChatGPT, with a focus on radiotherapy, ensuring accuracy, compliance, and continuous refinement.

Figure 4 illustrates the workflow for creating and maintaining an LLM-based medical chatbot powered by ChatGPT, tailored for radiotherapy. The chatbot relies on a curated database of verified information and undergoes domain-specific training to understand radiotherapy nuances. However, several limitations exist. The quality of training data and the effectiveness of cross-checking mechanisms are crucial; any errors or biases can lead to inaccurate responses. Regular updates are necessary to keep the database current, but this requires significant resources. User feedback helps refine the system, although the feedback can be subjective and challenging to manage. Error-handling mechanisms allow for query escalation, but not all issues may be resolved, potentially affecting user trust. Despite these challenges, the iterative process aims to provide accurate and up-to-date information within the chatbot’s expertise.

This process is crucial because it ensures the reliability, accuracy, and ethical integrity of the medical chatbot in radiotherapy. By curating a precise and verified database, fine-tuning the AI model, and implementing rigorous validation processes, the system can provide trustworthy information. The continuous refinement loop, fueled by user feedback and updates, ensures that the chatbot stays current with evolving practices and advancements in radiotherapy. This reliability and adherence to medical standards instills confidence in users, guiding them with accurate information while emphasizing the chatbot’s limitations and the necessity of seeking professional medical advice. Ultimately, this meticulous process not only educates and assists users but also upholds the ethical responsibility of providing reliable health care information through AI-driven technologies.

### Performance Evaluation

Evaluating the chatbot’s performance was a critical step to ensure that it met the high standards required for educational tools in radiotherapy. The evaluation focused on 3 main areas: accuracy, user satisfaction, and educational impact.

### Accuracy

The accuracy of the chatbot was assessed through a multitiered validation process. First, a set of standardized queries covering a wide range of radiotherapy topics was developed. These queries were designed to test the chatbot’s ability to provide correct and precise information. Subject matter experts in radiotherapy independently evaluated the chatbot’s responses to these queries, comparing them against established medical literature and clinical guidelines [79]. The chatbot’s performance was quantified using accuracy metrics such as precision and recall, ensuring that it consistently delivered reliable information [80]. In addition, the chatbot’s ability to update and incorporate the latest research findings was tested, emphasizing the need for dynamic content management to maintain long-term accuracy [81].

### User Satisfaction

User satisfaction was evaluated through extensive user testing, involving a diverse group of end users, including students, educators, and health care professionals. Surveys and feedback forms were used to gather qualitative and quantitative data on users’ experiences with the chatbot. Key aspects such as ease of use, response time, clarity of information, and overall user experience were measured. The feedback loop was integral to refining the chatbot, addressing any issues related to user interaction, and ensuring that the chatbot met the expectations of its target audience. In addition to surveys, usability testing sessions were conducted where users interacted with the chatbot in controlled environments, providing real-time feedback on their experiences. This iterative process helped in identifying and rectifying any usability challenges, enhancing the overall user satisfaction [79].

### Educational Impact

The educational impact of the chatbot was evaluated by analyzing its effectiveness as a learning tool in radiotherapy education. This involved conducting controlled studies where the performance of students who used the chatbot as a supplementary learning resource was compared to that of students who did not. Various educational metrics, such as knowledge retention, comprehension, and application of concepts, were measured through pre- and postintervention assessments [82]. The chatbot’s impact on these metrics was statistically analyzed to determine its effectiveness in enhancing learning outcomes [77]. Furthermore, longitudinal studies were conducted to assess the sustained educational benefits of the chatbot, ensuring that its use had a lasting positive impact on students’ understanding of radiotherapy concepts [83]. In addition to these formal evaluations, the chatbot’s performance was assessed in real-world educational settings through pilot studies. These studies provided insights into how the chatbot influenced classroom dynamics, student engagement, and the overall learning environment [84-86]. Feedback from these settings was crucial in understanding the practical implications of integrating the chatbot into existing curricula and further validated its educational impact.

### Lessons Learned and Future Directions

As the radiotherapy education chatbot continues to evolve, there are numerous opportunities for innovation that could significantly enhance its capabilities. Future research and technological advancements hold the potential to expand the chatbot’s functionality, improve user experiences, and further integrate it into the educational landscape.

### Advanced NLP and Understanding

One of the most promising areas for innovation lies in the continued development of advanced NLP algorithms. Future iterations of the chatbot could benefit from more sophisticated NLP techniques, such as transformer-based models and deep learning architectures, which can better understand and generate human-like responses [87]. These advancements would enable the chatbot to handle more complex queries, recognize nuanced language patterns, and provide more contextually accurate answers. Enhanced NLP capabilities could also improve the chatbot’s ability to engage in multiturn conversations, allowing for deeper and more meaningful interactions with users.

### Personalized Learning Experiences

Another key area for innovation is the development of personalized learning experiences. Leveraging data-driven insights, the chatbot could be equipped with adaptive learning algorithms that tailor content and responses to individual users’ needs, preferences, and learning paces [88]. By analyzing user interactions, the chatbot could identify areas where a student might be struggling and provide targeted educational resources or alternative explanations to enhance understanding. This personalized approach would make the chatbot a more effective tool for diverse learning styles and could significantly improve educational outcomes [89].

### Integration With Virtual and Augmented Reality

The integration of the chatbot with virtual and augmented reality (VR/AR) technologies presents exciting possibilities for creating immersive learning environments. By combining the chatbot’s informational capabilities with VR/AR platforms, users could engage in interactive simulations of radiotherapy procedures, enhancing their practical understanding of complex concepts [90,91]; for example, students could use the chatbot to guide them through virtual radiotherapy sessions, where they can visualize and manipulate different treatment parameters in a 3D space. This fusion of AI-driven education with immersive technologies could revolutionize how radiotherapy is taught and learned.

### Enhanced Multilingual and Cross-Cultural Capabilities

As radiotherapy education becomes increasingly globalized, there is a growing need for the chatbot to support multilingual and cross-cultural communication. Future developments could focus on expanding the chatbot’s language capabilities, enabling it to provide accurate and contextually relevant information in multiple languages. This would not only make the chatbot more accessible to non–English-speaking users but also allow it to adapt its responses to different cultural contexts, ensuring that the information is both accurate and culturally sensitive [92]. Such advancements would make the chatbot a valuable educational tool in diverse global settings.

### Ethical AI and Bias Mitigation

As AI continues to advance, addressing ethical concerns and mitigating biases in the chatbot’s responses will be paramount. Future research could explore more robust methods for ensuring that the chatbot’s algorithms remain unbiased and that its responses adhere to ethical guidelines [93]. This might include developing advanced algorithms for detecting and correcting biases in real time, as well as enhancing the transparency and explainability of the chatbot’s decision-making processes. By prioritizing ethical AI, the chatbot could set new standards for responsible AI use in education, fostering trust and reliability among users [20].

### Integration With Broader Educational Ecosystems

Finally, future innovations could focus on integrating the chatbot with broader educational ecosystems, including learning management systems and other digital educational tools [94,95]. By doing so, the chatbot could become a seamless part of the educational workflow, providing continuous support to students and educators across various platforms. This integration would enable more comprehensive data collection and analysis, allowing educators to monitor student progress in real time and make data-informed decisions to enhance teaching strategies. The chatbot could also facilitate collaborative learning by connecting users with peers, mentors, or experts, creating a more interactive and supportive educational community.

### Adaptability to Other Medical Fields

The framework developed for the radiotherapy education chatbot demonstrates significant potential for adaptation to other medical fields. By leveraging advanced NLP algorithms and personalized learning experiences, the chatbot can be tailored to provide specialized educational content across various medical disciplines, such as cardiology, neurology, and oncology. The integration of VR/AR technologies can further enhance learning by offering immersive simulations of medical procedures relevant to each field. Furthermore, the chatbot's multilingual and cross-cultural capabilities ensure that it can deliver accurate and contextually appropriate information to a diverse global audience. By addressing ethical AI considerations and integrating with broader educational ecosystems [18], the framework can support continuous learning and professional development for health care professionals worldwide, ultimately improving patient care and outcomes.

### Conclusions

This Viewpoint paper has explored the development of a resilient framework for a medical chatbot tailored to radiotherapy education, addressing key aspects such as accuracy, reliability, privacy, ethics, and the potential for innovation. The framework emphasizes the importance of maintaining up-to-date and accurate information, ensuring user trust through robust privacy measures, and fostering an ethical approach to AI in health care. Through performance evaluation, the chatbot demonstrated its capability to enhance learning outcomes and support health care professionals in their continuous education. Challenges such as bias, user engagement, and integration into existing systems were identified, with strategies proposed to overcome these obstacles.

Looking ahead, the future of LLM-based medical chatbots holds significant promise for radiotherapy education and health care as a whole. These technologies have the potential to revolutionize how complex medical knowledge is disseminated, making education more accessible, personalized, and interactive. By continuing to advance in areas such as NLP, personalized learning, and integration with immersive technologies, LLM-based chatbots can become indispensable tools in both educational and clinical settings. As these chatbots evolve, they will likely play a crucial role in shaping the future of health care, improving patient outcomes, and supporting the ongoing education of health care professionals worldwide. The framework presented in this paper serves as a foundational guide for the responsible and effective implementation of these powerful tools, ensuring that they contribute positively to the field of radiotherapy and beyond.

## Abbreviations

AI: artificial intelligence
LLM: large language model
NLP: natural language processing
VR/AR: virtual and augmented reality
